# Exploring the Association of Innate Immunity Biomarkers With MRI Features in Both Early and Late Stages Osteoarthritis

**DOI:** 10.3389/fmed.2020.554669

**Published:** 2020-11-12

**Authors:** Sureka Naidu Rajandran, Cheryl Ann Ma, Jin Rong Tan, Jin Liu, Steven Bak Siew Wong, Ying-Ying Leung

**Affiliations:** ^1^Department of Rheumatology & Immunology, Singapore General Hospital, Singapore, Singapore; ^2^Duke-NUS Medical School, Singapore, Singapore; ^3^Department of Diagnostic Radiology, Singapore General Hospital, Singapore, Singapore; ^4^Center for Quantitative Medicine, Duke-NUS Medical School, Singapore, Singapore; ^5^Department of Diagnostic Radiology, Seng Kang Hospital, Singapore, Singapore

**Keywords:** osteoarthritis, biomarkers, innate immunity, pro-inflammatory, magnetic resonance imaging

## Abstract

**Objective:** To evaluate the association between biomarkers of innate immunity and the magnetic resonance imaging (MRI) features of earlier and later stages of knee osteoarthritis (KOA).

**Methods:** From 139 and 20 participants with earlier and later stages of KOA, respectively, we analyzed knee MRIs scored using the Boston Leeds Osteoarthritis Knee Score (BLOKS) at recruitment with biomarkers. In paired serum (s) and synovial fluid (sf), we quantified three biomarkers related to innate immunity: lipopolysaccharide binding protein (LBP), CD14 and Toll-like receptor 4 (TLR4), and three proinflammatory biomarkers [interleukin-6 (IL6), IL8, and tumor necrosis factor alpha (TNFα)].

**Results:** In participants with earlier KOA, (s) LBP was statistically significantly associated with meniscal extrusion, and (sf) CD14 was associated with effusion after adjustment with age, sex, and body mass index. In participants with later stage of KOA, (sf) LBP was associated with effusion. (sf) CD14 was associated with cartilage loss and BML. In earlier stage of KOA, the proinflammatory biomarkers IL6, IL8, and TNFα were associated with most MRI features.

**Conclusion:** Innate immunity biomarkers (s) LBP was associated with MRI meniscal extrusion; (sf) CD14 was associated with MRI synovial inflammation in earlier stage and BMLs in later stage of KOA. Associations between proinflammatory biomarkers and various MRI features in earlier stage of KOA were observed.

## Introduction

Knee osteoarthritis (KOA) is the most common form of arthritis affecting about 250 million people worldwide and a leading cause of mobility and disability in the elderly (1, 2). Inflammation of the synovium may play a role in the pathophysiology of KOA, as evidenced by histological synovitis from the tissue harvested from patients undergoing arthroplasties (3–5). However, there is less information on earlier stages of KOA, and the pathophysiological characteristics of earlier KOA could be different. Greater knowledge of the initial stages of the disease may help advance understanding of KOA pathogenesis and identification of therapeutic targets for early KOA, which may be more responsive to treatment. Magnetic resonance imagining (MRI) is an imaging modality that reveals the pathology of all joint tissues including the cartilage, synovium, meniscus, and bone marrow. MRI can detect synovial membrane inflammation in early KOA even when signs of joint inflammation were not obvious in physical examination (6, 7); it therefore provides a mean for studying earlier stages of KOA.

It has been hypothesized that macrophage-associated innate inflammation may play a role in the pathogenesis of KOA (8, 9). Systemic lipopolysaccharide (LPS) levels were shown to be higher in obese animals, possibly related to increased intestinal permeability (10, 11). LPS, being a pathogen-associated molecular pattern (PAMP), binds to lipopolysaccharide binding protein (LBP) (12). The cluster of differentiation 14 (CD14) biomarker is predominantly found on activated macrophages and serves as a receptor for the LPS–LBP complex (9, 13). The binding of the LPS–LBP complex to CD14 would then trigger the Toll-like receptor 4 (TLR4) of macrophages (14, 15), leading to the downstream production of inflammatory mediators and catabolism of chondrocytes (16). Recently, both serum (s) and synovial fluid (sf) LPS and LBP were shown to be associated with the abundance of activated macrophages in the and synovium as well as associated with radiographic severity and symptoms among patients with KOA (17), suggesting the possible role of LPS in triggering of the innate immunity in KOA pathogenesis. TNFα and IL1β have been implicated as the key drivers of inflammatory cascade in KOA (16). IL6 was shown to be elevated in (s) and (sf) in KOA patients, stimulating metalloproteinase expression and mediating cartilage extracellular matrix protein degradation (18, 19). IL8 is an inflammatory chemokine mediating neutrophil accumulation and activating leukocyte homing to the synovium (16, 20).

In this study, we aim to evaluate the association of inflammatory biomarkers related to innate immunity: LBP, CD14, TLR4 with different MRI features of the knee from two studies of participants with KOA. These biomarkers represent the possible trigger, receptor, and effector cells (activated macrophage) of the innate immunity. We also evaluated the association of downstream inflammatory biomarkers: IL6, IL8, and TNFα with MRI features. Participants from one of studies represent earlier stage of KOA and the other with later stage of KOA.

## Materials and Methods

### Study Cohorts

We analyzed baseline clinical data and biological samples from two existing studies established in a tertiary hospital in Singapore.

### The Study With Earlier Stage of KOA

Participants were recruited from a cross-sectional study of 145 participants with knee pain. The study protocol was read and approved by the SingHealth Centralized Institutional Review Board (Ref: 2012/837/E), and informed consent were obtained from all participants prior to the start of the study. The participants (40–79 years old) from this study were recruited from the community via social media, advertisements, and referrals from the primary health care clinics (Singhealth Polyclinics) and two departments at the Singapore General Hospital (Rheumatology and Immunology; and Orthopedics) (21). Interested participants were invited to call through a recruitment telephone hotline and were then screened by trained staff. Participants who experienced pain in at least one knee on most days during the past month were invited to a screening clinic visit. All participants were examined by a rheumatologist (YYL) at the clinic. The study inclusion criteria included adult above 40 years old and a positive response to the question “Do you have pain, aching or stiffness of the knee on most days of the past month.” We excluded those who had prior knee replacement surgery or were planning for knee replacement surgery in the next 6 months. Radiography of both knees were taken in the clinic, and participants with Kellgren and Lawrence (KL) (22) grade 4 in either knee or isolated patellofemoral joint involvement on radiography as read by the attending rheumatologist were excluded. We excluded participants with significant joint injuries in the past 1 year and other joint diseases (rheumatoid arthritis, spondyloarthritis, Paget's disease, joint fractures, hyperparathyroidism, hyperthyroidism, hypothyroidism). We also excluded participants with contraindications to magnetic resonance imaging (MRI), such as significant renal impairment, pregnancy, metallic implants *in situ*, and claustrophobia. Participants using warfarin were excluded due to an increase risk of bleeding after joint aspiration. One hundred thirty-nine participants completed MRI of the knee. Sixty (43%) participants from this study fulfilled the American College of Rheumatology (ACR) Clinical and Radiographic Criteria for KOA (23). Among the 79 participants who did not fulfilled the ACR Clinical and Radiographic Criteria, 33 participants fulfilled classification criteria of early KOA suggested by Luyten et al. (24).

### The Study With Later Stage of KOA

We used the baseline data and biological samples from the Colchicine effectiveness in symptom and inflammation modification in knee osteoarthritis (COLKOA) (NCT02176460) (25, 26). The COLKOA is a double-blind, placebo-controlled, randomized trial that compared a 16-week treatment with a standard daily dose of oral colchicine to placebo for symptomatic KOA. The study protocol was read and approved by the SingHealth Centralized Institutional Review Board (CIRB2012/659/E), and informed consent was obtained from all participants before beginning the study. A total of 109 participants with symptomatic KOA based on the ACR Clinical and Radiographic Criteria (23) with KL grading of ≥2 in at least one knee during the screening were recruited in this study. We included in this study 20 participants who had MRI of the knee performed at baseline. Ten participants each from the colchicine and placebo arms were randomly selected for knee MRI according to a pre-specified list (25).

### Clinical Data Collection

In both studies, we collected age, sex, ethnicity, history of knee joint injury, comorbidities (hypertension, hyperlipidemia, diabetes mellitus, and coronary artery disease), and current medications. The body weight and height were measured using an electronic weight scale and an ultrasonic height sensor (Avamech B1000 Series) without shoes. The index knee was designated as the more symptomatic knee or the dominant knee if symptoms of both knees were similar. The clinical severity of KOA was evaluated using the Western Ontario and McMaster Universities Osteoarthritis Index (WOMAC) of the index knee (27). The WOMAC consists of 24 items that are divided into three subscales, which are pain, stiffness, and physical function. Each domain was standardized to obtain a score ranging from 0 to 100; higher scores reflected greater clinical severity.

### Radiographic Assessment

For both studies, all the participants underwent at recruitment posteroanterior fixed-flexion weight-bearing radiography on both knees with a SynaFlexer lower limb positioning frame (Synarc). The radiography was taken by starting with a 10° caudal beam angle until achieving alignment of the anterior and posterior margins of the tibial plateau to within 1.2 mm target (28). Each of the radiograph was scored according to the KL grading (0–4) of the tibiofemoral (TF) compartment (22) by an experienced musculoskeletal radiologist (SBW) who was blinded to the condition of the participants. A total of 70 radiographs (140 knees) from this study were rescored for KL grades by the original assessors 8–12 weeks apart and blinded to the original scores. The weighted kappa of KL grading of TF compartment was 0.60 [95% confidence interval (CI), 0.42–0.73].

### Magnetic Resonance Imaging Assessment

A total of 139 participants from the earlier KOA study and 20 participants from the later KOA study had MRI on their index knees (25). The MRI imaging were performed in a 45-min session by a 3-T Philips Ingenia machine with the index knee immobilized in a dedicated dStream T/R 16ch knee coil. The sequences assessed were coronal T1w, coronal proton density fat saturated, sagittal proton density fat saturated, axial T2w fat saturated, and axial T1w fat saturated (pre- and post-gadolinium contrast). The slice thickness was 2 mm, and the in-plane resolution matched the T1-weighted water excite scan with interpolation. The repetition time was 20 ms, and fat suppression (by water excitation) was utilized to minimize chemical shift artifacts.

The MRIs were assessed for osteophytes (OST) size, cartilage integrity, bone marrow lesions (BMLs), effusion, Hoff's synovitis, and meniscal extrusion according to the Boston Leeds Osteoarthritis Knee Score (BLOKS) scoring system (29) by a musculoskeletal radiologist (JRT) who was blinded to the participant's clinical details. OST size, cartilage integrity, BMLs, and meniscal extrusion scores were calculated according to the number of subregions affected (30). All 20 MRIs from this study of later stage of KOA were read twice by the same assessor blinded to the sequence of MRI and participants' condition. The weighted kappas of MRI scoring for BMLs (median, 0.68; range, 0.60–0.72), cartilage integrity (median, 0.73; range, 0.44–0.91), OST (median, 0.88; range, 0.87–0.90), and meniscal extrusion (median, 0.84; range, 0.82–0.85) were satisfactory, and the weighted kappa for effusion and Hoff's synovitis were 0.61 (95% CI, 0.36–0.85) and 0.76 (95% CI, 0.63–0.88), respectively.

### Biological Sample Collection

The protocol for biological sample collection and processing were standardized across both studies. Blood samples were collected at least 2 h postprandial from all the participants at recruitment. Knee synovial fluid was collected from all participants in the later KOA study and the earlier KOA study starting from the 41st participant onwards. The synovial fluid was aspirated directly from the index knee with a 20-gauge needle. In cases where no fluid was obtained, 10 ml of saline was injected into the joint and the fluid aspirated after gentle compression of the knee. Only participants with joint fluid obtained by direct aspiration was included in the current analyses (see below). The collected samples were centrifuged at 3,000 rpm for 15 min, aliquoted into separate vials, and stored in a −80°C freezer until analysis.

### Biomarker Assessment

The following (s) and (sf) biomarkers were measured with commercially available enzyme-linked immunosorbent assay (ELISA) kits for innate immunity, LBP, CD14, and TLR4, and downstream proinflammatory biomarkers, IL6, IL8, and TNFα. The measurements were performed according to the manufacturer's guidelines. (s) biomarkers were measured in duplicates to obtain the intra-assay coefficient of variations (CVs), except that (s) TLR4 assays were run in singlicate due to exhaustion of sample volume. (sf) biomarkers were measured in singlicate. To ensure reliability for samples measured in singlicate, we measured pooled (s) or (sf) samples in each plate. The pooled (s) or (sf) samples and kit standards were run in duplicate to derive the CVs. All intra- and interassay coefficients of variation (CVs) of the kit standards and sample controls were within 15% limits (Supplementary Table 1).

As majority of the biomarkers of the lavage samples from knee aspirate fell below the lower limit of detection (LLOD), we decided to limit the analysis of (sf) biomarkers to those samples obtained via direct aspiration. Therefore, the analyses of (sf) biomarkers were limited to 47 participants in the earlier KOA study and 14 participants in the later KOA study. Less than 70% of (s) IL6 and IL8 concentrations were above the lower limit of detection (LLOD) in the earlier KOA study; these biomarkers in the earlier KOA study were analyzed as a categorical variable (0 = concentrations below LLOD and 1 = concentrations above LLOD).

### Statistical Analysis

Majority of the biomarkers except (sf) and (s) IL6 and (s) IL8 were not normally distributed. Hence, we log transformed all biomarker data to achieve normality. We evaluated the associations between the (s) and (sf) biomarkers with MRI features, KL gradings, and WOMAC scoring using generalized linear models adjusted for age, sex, and body mass index (BMI). As MRI features and KL gradings were ordinal scales, the ordinal logistic model type was used within the generalized linear models for these outcomes. Since we tested for two items within the domains of cartilage loss and BMLs, we adjusted the *p* values using *Bonferroni's* correction and considered *p* < 0.025 as statistically significant to reduce the likelihood of type I errors. For all other domains, *p* < 0.05 were considered as statistically significant. The analyses were conducted using SPSS version 25 (IBM Corp.).

## Results

### Participants in Early KOA and Late KOA Cohort

The baseline characteristics of the 139 participants in earlier KOA study and 20 participants in later KOA study who had MRI data are summarized in Table 1. Characteristics of participants from both studies were similar to other KOA cohorts in terms of age, sex distribution, and BMI (31, 32).

**Table 1:** Population characteristics of participants from early and late KOA studies.

	**Earlier stage KOA study (*n* = 139)**	**Later stage KOA study with MRI data (*n* = 20)**
Age, years^¶^	55.5 ± 7.8	56.1 ± 6.5
Female (%)	82.5	65.0
BMI, kg/m^2¶^	26.0 ± 5.9	28.1 ± 4.8
**Ethnicity, n, (%)**		
Chinese	116 (81.1)	15 (75.0)
Malay	15 (10.5)	2 (10.0)
Indian	10 (7.0)	3 (15.0)
Others	2 (1.4)	0 (0)
**Comorbidities, n, (%)**		
Hypertension	43 (30.3)	7 (35.0)
Hyperlipidemia	55 (38.7)	7 (35.0)
Diabetes mellitus	12 (8.5)	1 (5.0)
Coronary artery disease	4 (2.8)	0 (0)
Duration of KOA, years^¶^	2.5 (4.9)	7.4 (7.1)
Fulfilled ACR Clinical and Radiographical Criteria for KOA (%)	43	100
**KL grade of index knee, n, (%)**		
0	40 (28.8)	1 (5.0)
1	44 (31.7)	5 (25.0)
2	35 (25.2)	6 (30.0)
3	18 (12.9)	5 (25.0)
4	2 (1.4)	3 (15.0)
WOMAC pain (0–100) ^¶^	31.1 ± 18.4	42.1 ± 21.1
WOMAC function (0–100) ^¶^	32.0 ± 19.9	44.9 ± 20.9
**Biomarkers^¥^**		
(s) LBP (ng/ml)	9079.0 (6745.0, 11794.0)	9466.5 (8072.3, 10514.3)
(sf) LBP (ng/ml)^£^	1796.9 (1448.5, 2416.4)	2012.9 (1579.6, 2315.6)
(s) TLR4 (pg/ml)	1049.0 (661.0, 1294.0)	699.5 (628.5, 1047.0)
(sf) TLR4 (pg/ml)^£^	875.2 (12.5, 1150.5)	3845.4 (3088.5, 4092.9)
(s) CD14 (ng/ml)	1254.8 (1145.0, 1374.7)	1327.5 (1223.8, 1555.5)
(sf) CD14 (ng/ml)^£^	255.3 (167.8, 400.8)	849.7 (681.5, 972.6)
(s) IL6 (pg/ml)	0.48 (0.48, 2.09)	3.35 (1.95, 5.65)
(sf) IL6 (pg/ml)^£^	87.45 (24.93, 248.06)	25.62 (12.43, 63.76)
(s) IL8 (pg/ml)	0.87 (0.32, 2.21)	4.95 (3.30, 9.15)
(sf) IL8 (pg/ml)^£^	5.09 (3.67, 9.22)	29.35 (24.02, 39.73)
(s) TNFα (pg/ml)	3.07 (1.10, 4.78)	4.75 (2.72, 5.66)
(sf) TNFα (pg/ml)^£^	1.62 (1.30, 1.90)	0.99 (0.74, 1.23)

*Knee osteoarthritis (KOA) was classified according to the American College of Rheumatology Clinical and Radiographic Criteria for KOA*.

¶ *Mean ± SD*.

¥ *Median (interquartile range)*.

£ *Limit to participants with direct aspiration of synovial fluid*.

*BMI, body mass index; KL grade, Kellgren and Lawrence grading; WOMAC, Western Ontario and McMaster Universities Osteoarthritis Index; (s), serum; (sf), synovial fluid; IL, interleukin; CD, cluster of differentiation; LBP, lipopolysaccharide binding protein; TNFα, tumor necrosis factor alpha; TLR4, Toll-like receptor 4*.

Compared participants in the study with earlier stage of KOA, participants in the study of later stage of KOA were older with a higher BMI and higher KL grade of the index knee. Participants in the late KOA cohort also had higher percentages of diabetes mellitus and hypertension. The duration of KOA was longer for participants in the later KOA study. The concentration of biomarkers for innate immunity and inflammation were generally higher in the later KOA study.

### Associations Between Synovial Fluid and Serum Biomarkers

Spearman's correlations between biomarkers are summarized in Supplementary Table 2. In both studies, (s) LBP correlated with the respective (sf) biomarkers. In the earlier KOA study, (s) and (sf) LBP correlated with proinflammatory markers IL6, IL8, and TNFα. (s) and (sf) TLR4 were correlated with each other in the earlier KOA study but not in the later KOA study. (s) and (sf) CD14 were not correlated with each other in both studies. In the later KOA study, (sf) CD14 was significantly correlated with (sf) IL6, IL8, and TNFα. The downstream proinflammatory biomarkers were generally correlated with each other.

### Associations Between Inflammatory Biomarkers With MRI Features and Other Outcomes

In the earlier KOA study, (s) LBP was associated with meniscal extrusion, and (sf) CD14 was associated with effusion (Table 2). These associations persisted after adjustment for age, sex, and BMI. The presence of downstream proinflammatory biomarkers (s) IL6, was associated with osteophytes, synovitis, effusion, and meniscus extrusion, while that of (sf) IL6 was associated with effusion. Similarly, (s) TNFα was statistically significantly associated with osteophytes, cartilage loss, synovitis, and effusion after adjustment.

**Table 2:** Association of inflammatory biomarkers with MRI and other features in the earlier stage of KOA, adjusted with age, gender, and BMI.

**Dependent variables**	**β** **(95% confidence intervals)**											
	**(s) LBP**	**(sf)** **LBP**	**(s)** **TLR4**	**(sf)** **TLR4**	**(s)** **CD14**	**(sf)** **CD14**	**(s)** **IL6^§^**	**(sf)** **IL6**	**(s)** **IL8^§^**	**(sf)** **IL8**	**(s)** **TNFα**	**(sf)** **TNFα**
OST size	−0.43 (−2.33, 1.47)	0.15 (−1.59, 1.90)	0.13 (−0.43, 0.69)	−0.15 (−0.80, 0.50)	−0.63 (−4.93, 3.67)	−0.75 (−1.77, 0.28)	**0.87** (0.21, 1.54)^**^^†^	−0.10 (−0.75, 0.55)	0.49 (−0.15, 1.13)	0.58 (−1.19, 2.35)	**0.91** (0.25, 7.21)^**^^†^	−2.30 (−4.61, 0.01)
% Cart loss	0.47 (−1.42, 2.37)	1.07 (−0.86, 3.00)	0.11 (−0.41, 0.64)	−0.30 (−0.97, 0.38)	1.64 (−2.63, 5.90)	−1.34 (−2.66, −0.01)^*^	0.64 (−0.01, 1.29)	−0.26 (−0.87, 0.36)	0.31 (−0.31, 0.93)	0.23 (−1.51, 1.97)	**0.83** (0.18, 1.49)^*^^†^	−2.78 (−5.23, −0.33)^*^
% Cart full thickness	−0.02 (−1.94, 1.91)	−0.19 (−2.16, 1.79)	0.52 (−0.04, 1.07)	0.40 (−0.28, 1.09)	0.97 (−3.30, 5.23)	−0.42 (−1.22, 0.37)	0.59 (−0.07, 1.25)	0.24 (−0.37, 0.85)	0.58 (−0.06, 1.23)	0.85 (−0.99, 2.69)	0.91 (0.24, 1.58)^**^^†^	−1.86 (−4.36, 0.64)
BML size	0.61 (−1.35, 2.56)	−0.13 (−1.81, 1.56)	0.38 (−0.15, 0.90)	0.05 (−0.61, 0.70)	0.73 (−3.51, 4.97)	−0.05 (−0.80, 0.69)	0.72 (0.06, 1.37)^*^	−0.08 (−0.74, 0.58)	0.55 (−0.09, 1.19)	1.01 (−0.88, 2.89)	0.73 (0.07, 1.38)^*^	0.36 (−1.82, 2.53)
% BML size	0.48 (−1.47, 2.44)	−0.14 (−1.83, 1.56)	0.33 (−0.20, 0.86)	0.11 (−0.54, 0.76)	1.08 (−3.17, 5.33)	−0.06 (−0.80, 0.69)	0.640 (−0.01, 1.29)	−0.07 (−0.73, 0.60)	0.59 (−0.05, 1.23)	1.06 (−0.84, 2.96)	0.71 (0.06, 1.37)^*^	0.44 (−1.73, 2.62)
Synovitis	1.38 (−0.75, 3.51)	1.03 (−0.79, 2.86)	0.49 (−0.18, 1.16)	0.06 (−0.59, 0.70)	2.93 (−1.90, 7.76)	0.71 (−0.18, 1.59)	**0.85** (0.17, 1.54)^*^^†^	0.66 (−0.10, 1.41)	0.69 (−0.00, 1.38)	1.21 (−0.80, 3.22)	**1.21** (0.44, 1.99)^**^^†^	0.07 (−2.56, 2.69)
Effusion	1.02 (−1.05, 3.08)	2.37 (−0.31, 5.05)	−0.19 (−0.74, 0.35)	0.20 (−0.51, 0.91)	2.45 (−2.22, 7.12)	**1.18** (0.123, 2.24)^*^^†^	0.50 (−0.18, 1.17)	**1.20** **(0.42, 1.98)** ^**^^†^	0.68 (0.01, 1.35)^*^	1.16 (−0.85, 3.18)	**0.93** (0.22, 1.64)^**^^†^	1.63 (−1.45, 4.72)
Meniscus extrusion	**3.10** (0.47, 5.74)^*^^†^	−0.10 (−2.18, 1.98)	−0.48 (−1.20, 0.25)	0.32 (−0.46, 1.09)	3.02 (−2.57, 8.61)	0.02 (−0.80, 0.83)	**0.87** (0.07, 1.67)^*^^†^	0.74 (−0.06, 1.54)	0.01 (−0.83, 0.85)	2.54 (−0.20, 5.28)	0.49 (−0.43, 1.42)	−0.61 (−3.25, 2.04)
KL grade	0.24 (−1.72, 2.19)	−0.30 (−2.25, 1.65)	0.47 (−0.13, 1.06)	0.50 (−0.18, 1.18)	2.32 (−2.11, 6.75)	0.10 (−0.67, 0.87)	0.53 (−0.14, 1.20)	0.10 (−0.59, 0.79)	0.19 (−0.45, 0.83)	−0.11 (−2.07, 1.85)	0.55 (−0.10, 1.20)	−2.03 (−4.67, 0.61)
WOMAC pain	3.51 (−16.32, 23.33)	−7.93 (−28.18, 12.32)	1.40 (−3.87, 6.66)	5.03 (−1.50, 11.55)	19.85 (−24.37, 64.06)	−0.70 (−9.89, 8.48)	−2.18 (−8.64, 4.27)	−3.57 (−9.67, 0.25)	2.06 (−4.21, 8.34)	−5.27 (−21.19, 10.67)	4.30 (−1.98, 10.58)	−13.71 (−35.84, 8.43)
WOMAC function	−11.85 (−32.72, 9.02)	−7.82 (−27.20, 11.57)	−0.50 (−6.06, 5.05)	5.23 (−0.90, 11.36)	13.24 (−33.38, 59.86)	−1.66 (−10.32, 7.00)	–**7.00** **(**–**13.72**, –0.25)^*^^†^	−3.17 (−8.94, 2.61)	0.89 (−5.74, 7.52)	−3.77 (−18.86, 11.31)	−2.28 (−8.92, 4.36)	−6.16 (−27.31, 14.99)

* *p < 0.05*,

** *p < 0.01*,

† *Bonferroni–adjusted p < 0.025 for cartilage loss and BMLs, p < 0.05 for all other domains (Bold)*.

§ *(s) IL6, IL8 and (sf) TLR4 were analyzed as categorical variable as more than 75% less than Lower limit of detection (0 = Below lower Limit of Detection and 1 = Above lower Limit of Detection)*.

*MRI, Magnetic Resonance Imaging; %, percentage; BML, Bone Marrow Lesion; Cart, Cartilage; OST, Osteophyte; KL grade, KL, Kellgren and Lawrence grading; (sf), synovial fluid; (s), serum; IL, interleukin; CD, cluster of differentiation; TNFα, Tumor Necrosis Factor alpha; LBP, Lipopolysaccharide Binding Protein; TLR, Toll–like receptor; vs., versus*.

None of the biomarkers related to innate immunity was associated with symptoms or radiographic gradings. The presence of (s) IL6 was negatively associated with WOMAC function that marginally reached statistical significance.

In the later KOA study, (sf) LBP was associated with effusion and WOMAC function. (sf) CD14 was associated with effusion in univariate analysis but lost statistical significance after adjustments (Supplementary Table 4). (sf) CD14 was associated with BMLs after adjustment of age, gender, and BMI (Table 3). None of the biomarkers related to innate immunity was associated with radiographic gradings. (s) IL6 was associated with WOMAC pain and function, while (sf) IL8 was associated with BMLs.

**Table 3:** Association of inflammatory biomarkers with MRI and other features in the later stage of KOA, adjusted with age, gender and BMI.

**Dependent variables**	**β** **(95% confidence intervals)**											
	**(s)** **LBP**	**(sf)** **LBP**	**(s)** **TLR4**	**(sf)** **TLR4**	**(s)** **CD14**	**(sf)** **CD14**	**(s)** **IL6**	**(sf)** **IL6**	**(s)** **IL8**	**(sf)** **IL8**	**(s)** **TNFα**	**(sf)** **TNFα**
OST size	3.30 (−8.98, 15.57)	11.33 (−3.60, 26.25)	−0.67 (−2.42, 1.09)	11.43 (−5.71, 28.57)	−4.67 (−20.86, 10.93)	4.41 (−5.30, 3.35)	0.42 (−1.44, 2.27)	−0.30 (−2.82, 2.22)	−2.37 (−5.43, 0.69)	6.72 (−5.37, 18.81)	−3.91 (−8.21, 0.38)	−2.69 (−14.48, 9.10)
% Cart loss	4.49 (−7.37, 16.36)	−24.84 (−1.75, 0.71)	−1.39 (−3.44, 0.65)	5.91 (−12.29, 24.10)	−12.87 (−30.91, 5.91)	2.08 (−0.26, 4.42)	0.59 (−1.85, 3.02)	−0.86 (−0.36, 1.89)	−3.50 (−7.48, 0.49)	6.64 (−5.94, 19.22)	−0.13 (−4.45, 4.18)	10.14 (−5.31, 25.58)
% Cart full thickness	4.69 (−7.34, 16.72)	17.01 (2.01, 32.05)^*^	−1.81 (−4.03, 0.40)	6.60 (−9.11, 22.31)	−15.84 (−33.03, 1.35)	2.79 (0.32, 5.25)^*^	0.51 (−1.91, 2.93)	−1.02 (−3.50, 1.46)	−1.84 (−5.05, 1.37)	6.21 (−4.65, 17.06)	−1.13 (−5.47, 3.21)	3.85 (−7.85, 15.55)
BML size	−0.15 (−11.06, 10.76)	0.38 (−8.92, 9.67)	−0.37 (−1.78, 1.04)	1.40 (−14.61, 17.41)	−5.88 (−23.49, 11.74)	**9.06** (1.62, 16.49)^*^^†^	0.99 (−1.05, 3.03)	0.58 (−1.52, 2.67)	−1.92 (−5.32, 1.48)	15.90 (1.06, 30.75)^*^	0.44 (−3.56, 4.44)	7.18 (−7.63, 21.99)
% BML size	1.16 (−10.39, 12.71)	1.84 (−7.24, 10.92)	−0.42 (−1.86, 1.02)	−2.6 (−18.28, 13.04)	−9.76 (−27.80, 8.27)	**9.06** (1.65, 16.48)^*^^†^	0.79 (−1.21, 2.80)	0.65 (−1.49, 2.79)	−2.04 (−5.51, 1.42)	**18.54** (3.68, 33.40)^*^^†^	−0.07 (−4.12, 3.98)	11.57 (−3.27, 26.30)
Synovitis	1.81 (−9.33, 12.94)	5.83 (−4.39, 16.04)	−0.32 (−1.92, 1.28)	8.92 (−9.73, 27.57)	3.14 (−14.58, 20.86)	1.74 (−0.55, 4.03)	−0.60 (−2.86, 1.66)	−0.43 (−2.74, 1.88)	−3.04 (−6.67, 0.58)	8.13 (−3.93, 20.19)	1.54 (−2.74, 5.82)	7.02 (−6.34, 20.37)
Effusion	13.87 (−0.08, 27.81)	**14.07** (0.49, 27.66)^*^	−0.47 (−1.97, 1.05)	1.17 (−16.16, 18.50)	−10.57 (−28.29, 7.15)	8.25 (−12.66, 29.16)	0.46 (−1.60, 2.52)	0.07 (−2.49, 2.63)	−0.75 (−3.74, 2.25)	2.53 (−8.47, 13.53)	−0.30 (−4.47, 3.88)	15.91 (−1.67, 33.48)
Meniscus extrusion	3.02 (−9.65, 15.68)	7.32 (−2.91, 17.54)	−0.71 (−2.28, 0.86)	−5.70 (−20.36, 8.97)	−12.80 (−30.09, 4.48)	1.55 (−0.90, 4.00)	−0.60 (−2.68, 1.49)	−0.20 (−1.74, 2.40)	−2.82 (−6.16, 0.51)	2.56 (−7.95, 13.08)	−2.30 (−6.43, 1.83)	1.53 (−9.71, 12.77)
KL grade	2.84 (−8.17, 13.85)	−4.21 (−14.43, 6.01)	−0.62 (−2.19, 0.96)	−12.38 (−31.86, 7.09)	−9.04 (−26.06, 7.97)	2.19 (−0.77, 5.14)	0.52 (−1.53, 2.57)	343.7 (−38,869, 39,556)	−1.47 (−4.77, 1.83)	7.31 (−4.19, 18.81)	−0.83 (−4.92, 3.27)	8.96 (−5.32, 23.23)
WOMAC pain	88.40 (−9.30, 186.1)	−1.47 (−3.94, 1.00)	−3.58 (−18.60, 11.43)	27.26 (−102.1, 156.7)	–**148.8** **(**–**290.4**, –7.13)^*^^†^	9.72 (−5.70, 26.15)	**26.91** (10.63, 43.20)^**^^†^	−2.43 (−20.22, 15.37)	−16.45 (−44.92, 12.02)	−10.98 (−62.86, 40.90)	−8.05 (−46.38, 30.28)	26.54 (−44.63, 97.71)
WOMAC function	71.45 (−34.24, 177.15)	**97.60** (29.88, 165.3)^**^^†^	−0.58 (−16.41, 15.25)	77.64 (−67.94, 223.2)	−66.05 (−227.0, 94.93)	−4.05 (−22.16, 14.05)	**25.50** (7.62, 43.39)^**^^†^	−8.54 (−28.05, 10.98)	−10.12 (−40.60, 20.37)	−35.14 (−90.75, 20.48)	−16.17 (−55.92, 23.58)	−2.92 (-84.29, 78.45)

* *p < 0.05*,

** *p < 0.01*.

† *Bonferroni-adjusted p < 0.025 for cartilage loss and BMLs, p < 0.05 for all other domains bold)*.

*MRI, magnetic resonance imaging; %, percentage; BML, bone marrow lesion; Cart, cartilage; OST, osteophyte; KL grade, Kellgren and Lawrence grading; (sf) synovial fluid; (s), serum; IL, interleukin; CD, cluster of differentiation; TNFα, tumor necrosis factor alpha; LBP, lipopolysaccharide binding protein; TLR, Toll-like receptor; vs., versus*.

## Discussion

Our study has demonstrated that biomarkers of innate immunity were associated with MRI features in both earlier and later stages of KOA. (sf) CD14 was positively associated with effusion on MRIs before and after adjustment for age, sex, and BMI in the earlier stage of KOA. In addition, (sf) CD14 was associated with BMLs in the later stage of KOA. Association between (s) LBP and meniscal extrusion was noted only in earlier stage KOA. In the earlier stage of KOA, the associations of a wider spectrum of downstream proinflammatory biomarkers with various MRI features were more prominently observed. In general, higher concentrations of all downstream proinflammatory biomarkers were observed in the participants with later stage of KOA than those with earlier stage of KOA.

Inflammation of the synovium has been observed in early KOA via synovial biopsies and MRI even before radiographical changes occurred (4, 33). There is growing evidence that synovitis is implicated in the symptoms and disease progression of KOA (34–37). Immunohistochemical studies have demonstrated that synovial tissue has a mononuclear cell infiltrate, particularly in early KOA (35, 38). This is consistent with our data that showed an association with (sf) CD14 and effusion in earlier stages of KOA. Soluble (sf) CD14 is a cellular marker that is predominantly found on activated macrophages and monocytes (39). Daghestani et al. have previously shown a positive association between baseline macrophage markers (sf) CD14 and (sf) and (s) CD163 with the abundance of activated macrophages in the synovium (9). These biomarkers were also associated with the radiographic progression of KOA after 3 years. This suggested that macrophage-related synovitis could be a driver of the structural damage in KOA and its progression. This is in line with murine models that have shown that synovial macrophages were involved in osteophyte formation and growth (40). Our current findings concur with the hypothesis that macrophages were associated with signs of inflammation (effusion) for the earlier stage of KOA, while (sf) CD14 was associated with BMLs in the later stage of KOA. The postulated upstream trigger, (sf) LBP, was associated with effusion in the later stage of KOA. In both studies, we did not observe an association of synovitis with innate immunity biomarkers. One possible explanation is that the synovitis scoring used in this study measures inflammation in the infra-patella fat pad, which may not be as in close relation to effusion or special imaging for activated macrophages used in other study (17, 41). In the later stage of KOA, (sf) CD14 continued to associate with a wider spectrum of MRI features in different structures, including BMLs and marginally significant for cartilage loss. This suggested that innate immunity plays an important role in pathogenesis in different stages of KOA and may be particularly important in driving inflammation in the synovium and the downstream proinflammatory response in earlier KOA.

In the current study, we found that (s) LBP, which is a protein that binds to the LPS, was positively associated with meniscal extrusion on MRI in the earlier stage of KOA, while (sf) LBP was associated with effusion and WOMAC function in the later stage of KOA. It has been hypothesized that circulating lipopolysaccharide (LPS) may activate the synovial macrophages and contribute to the pathogenesis of OA (14). Studies have shown that systemic levels of LPS are elevated in obese patients even in the absence of an infection. Both animal and human studies have shown that obesity is linked to intestinal permeability (10–12). In a healthy environment, the intestinal mucosa provides a selectively permeable membrane between the systemic circulation and the intestinal lumen (13). A high-fat diet, however, disrupts the expression and localization of the tight junction protein in the small intestine that results in an increased permeability of the intestine (10, 42) and causes the LPS in the gut to be released in the systemic circulation. The LPS in the systemic circulation binds to LBP and may subsequently activate the macrophage by binding to CD14 and TLR4 (13). The activation of the macrophage can result in the synthesis of proinflammatory cytokines such as IL6, IL8, and TNFα, leading to degeneration of articular cartilage (16, 43). Huang et al. have previously shown that both (s) and (sf) LPS and LBP in KOA patients were associated with the abundance of activated macrophages in the synovium, radiographic severity, and KOA symptoms (17). In another study, the change in (s) LPS/LBP over 12–18 months were associated with radiographic progression of KOA, while the change in (s) TLR4 over 18 months was associated with cartilage degradation marker (CTXII) (44). The finding of our study therefore concurs with the role of LPS/LBP pathways in association with the innate immunity in the pathogenesis of KOA.

Our study found that the associations between the downstream proinflammatory biomarkers with MRI-detected BMLs and synovial inflammation were more prominently seen in the earlier stage of KOA as compared to the later stage of KOA. A greater intensity of inflammation in OA during the early phase has been described in other studies (35, 38, 45). Although inflammatory biomarkers were found in all stage of KOA, Smith at al. have shown a decrease in the ratio of IL1 receptor antagonist to IL1α and β with increasing radiographic grades of KOA (38). Benito et al. have demonstrated a significantly greater CD4+ and CD68+ cell infiltration, number of cells producing TNFα and IL1β, together with greater blood vessel formation, vascular endothelial growth factor, and intercellular adhesion molecule-1 expression in the synovial tissue taken from participants undergoing arthroscopy for knee pain compared to those undergoing knee replacement surgery for late-stage KOA (35). Similarly, Ene et al. have observed a more extensive synovitis with mononuclear and macrophage infiltrates, diffused fibrosis, thickening of the lining layer, and neovascular formation in participants with earlier KOA who underwent arthroscopy compared to those with end-stage KOA who underwent knee replacement surgery (45). One possible reason that explains the relative less associations we observed between proinflammatory biomarkers and MRI features in the later stage of KOA in the current study lies in the semiquantitative MRI scoring system. The number of subregions affected introduces substantial ceiling effect in the later stage of KOA, which limited the capacity of showing association with increasing levels of proinflammatory biomarkers. Taken together, our study supports a prominent role of inflammation particularly in earlier stage of KOA.

The strength of our study is having two studies at different stages of KOA. Paired (s) and (sf) samples were collected together with MRI imaging that provided detailed assessment of various tissue structure of the knee as compared to radiography. The concentration of the measured biomarkers was consistent with other studies (17, 44). There are a few limitations in the interpretation for our study. The cross-sectional study design showed associations rather than causal relationship between the biomarkers and KOA. The associations may give insight into the pathogenesis of KOA, but the study design does not allow evaluation of pathogenic mechanisms. The associations between various biomarkers were not readily interpretable clinically. Further research is required for the validation of current findings. Nonetheless, it provides evidence to support the role of innate immunity in all stages of KOA. The sample size of the study in the later stage of KOA was small and may result in limited statistical power to demonstrate small associations. These results need to be validated with a larger cohort. The (sf) analyses were only limited to participants whose joint fluid was obtained via direct aspiration, as majority of biomarker concentrations from lavage samples fall in undetectable ranges. Hence, the results for (sf) may only apply to participants with more joint effusion. We recognize that the classification of earlier and later stages of KOA was arbitrary. In the continuum of progression of chronic illness like KOA, a cutoff between early and late stage would be difficult, and there is currently no standardized definition for early KOA. With the recruitment criteria in these studies, one with knee pain in the past 1 month while another seeking active treatment, we believe that participants from the two studies generally represented different stages KOA, and this is also reflected by the lower KL scoring in the index knees and shorter duration of illness in the earlier KOA studies. Associations of biomarkers with MRI features in either study were performed separately with adjustment for only age, sex, and BMI. We did not perform any multivariable analysis, and no formal statistical comparisons between studies have been made. Therefore, the study can only be considered exploratory, which gives preliminary insight into different stages of KOA. In addition, the recruitment was based on clinical justifications with assistive radiographies of both knees read by the attending rheumatologist, which resulted in 1.4% of participants in the study of earlier KOA who had KL = 4 and 30% of participants in study of the later KOA who had KL <2 in the index knee as read by the radiologist. Lastly, participants in both studies had KOA and persistent pain; no comparison of the biomarker concentrations with healthy controls has been conducted in the current study.

In conclusion, the present exploratory study supports the association between biomarkers of activated macrophages and synovial inflammation in the earlier stage of KOA. This may be related to activation of the innate immunity via LPS and LBP. In addition to synovial inflammation, innate immunity activities were associated with BMLs and cartilage loss in the later stage of KOA. This suggests the important role of innate immunity in different KOA stages.

## Data Availability Statement

The raw data supporting the conclusions of this article will be made available by the authors, without undue reservation.

## Ethics Statement

The studies involving human participants were reviewed and approved by The SingHealth Centralized Institutional Review Board E. The patients/participants provided their written informed consent to participate in this study.

## Author Contributions

Y-YL and CAM conceptualized and designed the study. SR, CAM, JRT, SW, and Y-YL acquired the data. SR, Y-YL, and JL performed the data analysis. SR and Y-YL drafted the manuscript. All authors interpreted the data, critically revised the manuscript, and approved the final version of manuscript.

## Conflict of Interest

The authors declare that the research was conducted in the absence of any commercial or financial relationships that could be construed as a potential conflict of interest.
