# Supplementary material for: Exploring the Association of Innate Immunity Biomarkers With MRI Features in Both Early and Late Stages Osteoarthritis
Source: Front Med (Lausanne). 2020 Nov 12;7:554669. doi: 10.3389/fmed.2020.554669 (PMC7689194; doi:10.3389/fmed.2020.554669)
Supplement: Supplementary file 1 [file Data_Sheet_1.pdf]

Online supplementary material

Rajandran SN et al. Exploring the association of innate immunity biomarkers with MRI features in both early and late stages osteoarthritis

Supplementary Table I. Assay information of all measured biomarkers

Biomarkers	Biomarker Function	LLOD	Manufacturer	Serum				Synovial fluid				Mean intra assay CV (%)	Mean inter assay CV (%)
				Number of samples measured		Samples with concentration above LLOD (%)		Number of samples measured		Samples with concentration above LLOD (%)			
				Early KOA	Late KOA	Early KOA	Late KOA	Early KOA	Late KOA	Early KOA	Late KOA		
LBP	Bind to LPS to elicit immune response	4.4ng/mL	Hycult Biotech	145	109	99.3	99.1	47	69	97.9	98.6	3.8	4.0
TLR4	Transmembrane protein that detects PAMPs	25pg/mL	Arigo Biolab	145	109	91	88.1	47	69	70.8$^{\delta}$	100	3.5	5.0
CD14	Marker on activated macrophages	125pg/mL	R&D Systems	145	109	100	100	47	69	97.9	100	2.3	3.3
IL6	Pro-inflammatory cytokine	0.5pg/mL	Merck	145	109	48.3$^{\delta}$	100	47	69	97.9	100	5.7	5.9
IL8	Chemokine produced by macrophages	0.5pg/mL	Merck	145	109	64.8$^{\delta}$	100	47	69	97.9	100	5.7	5.9
TNFα	Marker of systemic inflammation	0.5pg/mL	Merck	145	109	81.3	100	47	69	91.7	100	5.7	5.9

$^{\delta}$ Biomarkers analysed as categorical variables (0 = concentration below LLOD, 1 = concentration above LLOD). IL = Interleukin; CD = Cluster of differentiation; TNFα = Tumour necrosis factor alpha; LBP = Lipopolysaccharide binding protein; LPS = Lipopolysaccharide; TLR4 = Toll-like receptor 4; PAMPs = Pathogen associated molecular patterns; CV = Coefficient of Variation; LLOD = Lower limit of detection; Early = Early KOA cohort; Late = Late KOA cohort.

Supplementary Table II. Spearman's Rho Correlations between various biomarkers

Earlier stage of KOA

	(s) LBP	(sf) LBP	(s) TLR4	(sf) TLR4	(s) CD14	(sf) CD14	(s) IL6	(sf) IL6	(s) IL8	(sf) IL8	(s) TNFα	(sf) TNFα
(s) LBP	-	0.395**	0.029	0.183	0.166	-0.142	0.200*	0.238	0.074	0.459**	0.278**	0.352*
(sf) LBP	0.395**	-	-0.092	-0.012	0.113	0.160	0.280	0.487**	0.280	0.324*	0.042	0.475**
(s) TLR4	0.029	-0.092	-	0.384**	-0.014	0.079	0.010	0.022	0.029	-0.144	-0.004	-0.029
(sf) TLR4	0.183	-0.012	0.384**	-	-0.142	-0.040	0.058	0.081	0.017	0.003	-0.074	0.161
(s) CD14	0.166	0.113	-0.014	-0.142	-	0.211	0.046	0.337*	0.083	0.261	0.030	-0.037
(sf) CD14	-0.142	0.160	0.079	-0.040	0.211	-	0.014	0.249	-0.031	0.100	-0.170	-0.086
(s) IL6	0.200*	0.280	0.010	0.058	0.046	0.014	-	0.199	0.461**	0.426**	0.565**	0.371**
(sf) IL6	0.238	0.487**	0.022	0.081	0.337*	0.249	0.199	-	0.144	0.510**	0.129	0.444**
(s) IL8	0.074	0.280	0.029	0.017	0.083	-0.031	0.461**	0.144	-	0.155	0.531**	0.191
(sf) IL8	0.459**	0.324*	-0.144	0.003	0.261	0.100	0.426**	0.510**	0.155	-	0.205	0.644**
(s) TNFα	0.278**	0.042	-0.004	-0.074	0.030	-0.170	0.565**	0.129	0.531**	0.205	-	0.278
(sf) TNFα	0.352*	0.475**	-0.029	0.161	-0.037	-0.086	0.371**	0.444**	0.191	0.644**	0.278	-

Later stage of KOA

	(s) LBP	(sf) LBP	(s) TLR4	(sf) TLR4	(s) CD14	(sf) CD14	(s) IL6	(sf) IL6	(s) IL8	(sf) IL8	(s) TNFα	(sf) TNFα
(s) LBP	-	0.741**	0.040	-0.357	-0.003	0.269	0.309	0.402	0.038	0.162	0.059	0.408
(sf) LBP	0.741**	-	-0.210	-0.238	-0.189	0.378	0.274	-0.119	-0.504	0.217	0.105	0.410
(s) TLR4	0.040	-0.210	-	0.483	-0.530*	0.123	0.082	0.147	0.196	0.002	0.010	0.262
(sf) TLR4	-0.357	-0.238	0.483	-	-0.154	-0.448	-0.288	-0.455	0.417	-0.476	0.343	-0.378
(s) CD14	-0.003	-0.189	-0.530*	-0.154	-	-0.140	-0.282	0.115	0.175	0.012	0.079	-0.234
(sf) CD14	0.269	0.378	0.123	-0.448	-0.140	-	0.106	0.487*	-0.360	0.574**	-0.127	0.762**
(s) IL6	0.309	0.274	0.082	-0.288	-0.282	0.106	-	0.365	-0.274	-0.066	0.118	0.374
(sf) IL6	0.402	-0.119	0.147	-0.455	0.115	0.487*	0.365	-	-0.113	0.361	-0.162	0.612**
(s) IL8	0.038	-0.504	0.196	0.417	0.175	-0.360	-0.274	-0.113	-	-0.266	0.228	-0.334
(sf) IL8	0.162	0.217	0.002	-0.476	0.012	0.574**	-0.066	0.361	-0.266	-	-0.059	0.542*
(s) TNFα	0.059	0.105	0.010	0.343	0.079	-0.127	0.118	-0.162	0.228	-0.059	-	0.041
(sf) TNFα	0.408	0.410	0.262	-0.378	-0.234	0.762**	0.374	0.612**	-0.334	0.542*	0.041	-

*$p<0.05$, **$p<0.01$; (sf) = synovial fluid; (s) = serum; LBP = Lipopolysaccharide Binding Protein; TLR = Toll-like receptor; IL= interleukin; CD = cluster of differentiation; TNFα = Tumour Necrosis Factor alpha;

Supplementary Table III. Univariate associations between biomarkers and MRI features and other outcomes in earlier stage of KOA

Dependent variables	β (95% confidence intervals)											
	(s) LBP	(sf) LBP	(s) TLR4	(sf) TLR4[§]	(s) CD14	(sf) CD14	(s) IL6[§]	(sf) IL6	(s) IL8[§]	(sf) IL8	(s) TNFα	(sf) TNFα
OST Size	1.21 (-0.67, 2.98)	0.29 (-1.48, 2.06)	0.28 (-0.24, 0.79)	-0.05 (-0.63, 0.53)	0.82 (-3.29, 4.92)	-0.60 (-1.58, 0.38)	**1.41 (0.77, 18.93)**†**	-0.00 (-0.64, 0.64)	**0.71 (0.09, 1.34)*†**	1.49 (-0.05, 3.02)	**1.23 (0.61, 1.86)**†**	-1.38 (-3.56, 0.80)
% Cart Loss	1.34 (-0.43, 3.11)	0.68 (-1.11, 2.47)	0.34 (-0.16, 0.84)	-0.41 (-1.01, 0.20)	3.24 (-1.01, 7.49)	-1.35 (-2.60, -0.10)*	**1.03 (0.41, 1.64)**†**	-0.16 (-0.74, 0.42)	0.55 (-0.07, 1.17)	0.67 (-0.86, 2.20)	**1.14 (0.52, 1.76)**†**	-2.02 (-4.40, 0.36)
% Cart Full Thickness	1.17 (-0.62, 2.96)	-0.23 (-2.18, 1.73)	**0.65 (0.12, 1.18)*†**	1.73 (-0.42, 0.76)	2.38 (-1.80, 6.56)	-0.46 (-1.24, 0.31)	**1.04 (0.42, 1.65)**†**	0.27 (-0.31, 0.86)	**0.85 (0.22, 1.48)**†**	1.16 (-0.57, 2.89)	**1.23 (0.59, 1.86)**†**	-1.17 (=3.66, 1.32)
BML Size	1.21 (-0.62, 3.05)	-0.01 (-1.68, 1.65)	0.51 (0.02, 1.01)*	0.07 (-0.49, 0.64)	1.69 (-2.36, 5.73)	-0.05 (-0.78, 0.68)	**0.99 (0.38, 1.61)**†**	-0.05 (-0.69, 0.59)	**0.74 (0.11 1.36)*†**	1.29 (-0.70, 3.28)	**0.98 (0.34, 1.61)**†**	0.98 (-1.08, 3.05)
% BML Size	1.12 (-0.72, 2.96)	-0.01 (-1.68, 1.66)	0.47 (-0.03, 0.97)	0.12 (-0.45, 0.68)	1.94 (-2.13, 6.00)	-0.05 (-0.77, 0.68)	**0.91 (0.30, 1.52)**†**	-0.04 (-0.69, 0.61)	**0.75 (0.12, 1.38)*†**	1.31 (-0.71, 3.32)	**0.95 (0.32, 1.59)**†**	1.04 (-1.03, 3.11)
Synovitis	1.70 (-0.33, 3.73)	0.68 (-1.05, 2.41)	0.54 (-0.11, 1.19)	-0.10 (-0.66, 0.46)	3.17 (-1.41, 7.74)	0.59 (-0.27, 1.44)	**0.98 (0.32, 1.63)**†**	0.55 (-0.15, 1.25)	**0.75 (0.07, 1.43)*†**	0.83 (-0.94, 2.59)	**1.27 (0.52, 2.02)**†**	-0.85 (-3.27, 1.57)
Effusion	1.74 (-0.18, 3.66)	1.98 (-0.54, 4.51)	-0.01 (-0.53, 0.52)	-0.03 (0.64, 0.57)	2.47 (-1.88, 6.83)	**1.06 (0.02, 2.10)*†**	**0.79 (0.15, 1.43)*†**	**1.17 (0.40, 1.94)**†**	**0.82 (0.17, 1.47)*†**	1.16 (-0.65, 2.98)	**1.21 (0.53, 1.88)**†**	1.05 (-1.66, 3.75)
Meniscus Extrusion	**4.47 (2.06, 6.89)**†**	0.31 (-1.68, 2.30)	-0.00 (-0.58, 0.58)	0.08 (-0.51, 0.67)	2.50 (-2.24, 7.23)	0.04 (-0.72, 0.80)	**1.27 (0.56, 1.98)**†**	**0.84 (0.12, 1.56)***	0.39 (-0.34, 1.12)	**3.93 (1.27, 6.58)**†**	**1.07 (0.25, 1.90)*†**	0.40 (-1.99, 2.79)
KL grade	1.13 (-0.70, 2.96)	-0.32 (-2.22, 1.58)	0.56 (-0.01, 1.12)	0.13 (-0.43, 0.69)	2.93 (-1.27, 7.13)	0.04 (-0.71, 0.80)	**0.93 (0.30, 1.56)**†**	0.17 (-0.49, 0.82)	0.38 (-0.24, 1.01)	0.33 (-1.43, 2.10)	**0.85 (0.23, 1.46)**†**	-1.43 (-3.84, 0.98)
WOMAC pain	4.86 (-14.07, 23.78)	-9.32 (-29.66, 11.02)	1.54 (-3.72, 6.80)	2.81 (-3.06, 8.69)	24.57 (-17.93, 67.06)	-1.38 (-10.61, 7.84)	0.62 (-5.59, 6.83)	-3.16 (-9.25, 2.92)	2.81 (-3.49, 9.10)	-5.72 (-20.75, 9.30)	5.08 (-1.04, 11.20)	-12.05 (-33.68, 9.58)
WOMAC function	-4.02 (-24.46, 16.43)	-7.55 (-27.84, 12.74)	0.03 (-5.65, 5.72)	2.57 (-3.25, 8.39)	19.53 (-26.39, 65.44)	-2.00 (-11.10, 7.11)	-2.07 (-8.79, 4.65)	-1.70 (-7.76, 4.35)	2.22 (-4.59, 9.03)	0.64 (-14.30, 15.58)	0.37 (-6.30, 7.04)	0.30 (-21.32, 21.92)

**p<0.05, **p<0.01, † Bonferroni-adjusted p<0.025 for cartilage loss and BMLs, p<0.05 for all other domains (Bold).*

[§] (s) IL6 and IL8 analysed as categorical variable in the early KOA cohort as more than 70% less than Lower limit of detection (0 = Below Lower Limit of Detection and 1 = Above Lower Limit of Detection).

MRI = Magnetic Resonance Imaging; % = percentage; BML = Bone Marrow Lesion; Cart = Cartilage; OST = Osteophyte; KL = Kellgren and Lawrence grading; (sf) = synovial fluid; (s) = serum; IL= interleukin; CD = cluster of differentiation; TNFα = Tumour Necrosis Factor alpha; LBP = Lipopolysaccharide Binding Protein; TLR = Toll-like receptor; vs.=versus.

Supplementary Table IV. Univariate associations between biomarkers and MRI features and other outcomes in later stage of KOA

Dependent variables	β (95% confidence intervals)											
	(s) LBP	(sf) LBP	(s) TLR4	(sf) TLR4	(s) CD14	(sf) CD14	(s) IL6	(sf) IL6	(s) IL8	(sf) IL8	(s) TNFα	(sf) TNFα
OST Size	6.09 (-3.68, 15.86)	7.87 (-2.87, 18.61)	0.04 (-1.36, 1.43)	7.59 (-7.23, 22.41)	-3.69 (-17.41, 10.04)	0.90 (-0.71, 2.50)	0.51 (-1.27, 2.29)	-0.13 (-2.44, 2.19)	-1.16 (-3.66, 1.34)	5.96 (-5.09, 17.00)	-2.83 (-6.77, 1.11)	-1.12 (-11.18, 8.94)
Cart Loss %	5.58 (-3.88, 15.03)	15.29 (0.82, 29.77)*	-0.78 (-2.55, 1.00)	1.81 (-14.03, 17.64)	-8.98 (-25.00, 7.05)	1.56 (-0.37, 3.49)	0.82 (-1.53, 3.17)	-0.52 (-2.69, 1.64)	-2.11 (-5.25, 1.03)	4.12 (-7.04, 15.28)	-0.23 (-4.26, 3.80)	6.84 (-5.41, 19.09)
% Cart Full Thickness	5.66 (-3.91, 15.22)	12.11 (0.21, 24.02)*	-1.18 (-2.30, 0.64)	2.34 (-11.93, 16.60)	-11.52 (-27.09, 4.05)	1.98 (-0.07, 4.02)	0.66 (-1.63, 2.95)	-0.57 (-2.65, 1.50)	-1.15 (-4.06, 1.77)	4.55 (-5.44, 14.54)	-1.29 (-5.28, 2.70)	3.79 (-6.62, 14.20)
BML Size	-2.35 (-10.98, 6.27)	-1.03 (-9.09, 7.02)	-0.49 (-1.61, 0.67)	-0.99 (-17.11, 11.12)	-1.01 (-16.48, 14.46)	1.59 (-0.39, 3.59)	0.33 (-1.63, 2.30)	0.60 (-1.33, 2.52)	-1.35 (-4.06, 1.35)	4.54 (-5.27, 14.35)	0.05 (-3.40, 3.51)	-1.04 (-10.51, 8.45)
% BML Size	-2.33 (-11.06, 6.41)	-0.52 (-8.38, 7.34)	-0.50 (-1.66, 0.65)	-3.84 (-17.81, 10.14)	-1.85 (-17.27, 13.56)	2.16 (-0.04, 4.36)	0.08 (-1.81, 1.98)	0.60 (-1.33, 2.52)	-1.38 (-4.07, 1.31)	5.12 (-4.74, 14.97)	-0.18 (-3.64, 3.28)	-0.61 (-10.02, 8.81)
Synovitis	5.53 (-3.74, 14.79)	4.66 (-4.62, 13.94)	-0.05 (-1.37, 1.27)	6.64 (-10.65, 23.92)	3.97 (-12.08, 20.04)	1.17 (-0.78, 3.13)	-0.14 (-2.16, 1.87)	-0.16 (-2.29, 1.97)	-1.26 (-4.18, 1.65)	7.37 (-3.89, 18.63)	0.54 (-3.17, 4.25)	6.28 (-5.09, 17.64)
Effusion	3.24 (-5.08, 11.56)	8.73 (-1.01, 18.47)	0.50 (-0.77, 1.77)	6.08 (-7.78, 19.93)	**-16.69 (-32.17, -1.21)*†**	4.19 (-2.33, 10.72)	0.70 (-1.12, 2.52)	-0.88 (-2.87, 1.11)	-1.52 (-4.15, 1.11)	0.51 (-9.93, 10.95)	0.60 (-3.15, 4.36)	1.17 (-8.26, 10.60)
Meniscus Extrusion	1.89 (-8.36, 12.13)	4.94 (-4.05, 13.94)	-0.47 (-0.69, 0.74)	-4.28 (-17.47, 8.91)	-4.98 (-18.72, 8.77)	1.24 (-0.64, 3.12)	-0.86 (-2.82, 1.09)	0.15 (-1.77, 2.08)	-1.64 (-4.36, 1.08)	0.48 (-9.14, 10.10)	-2.37 (-6.13, 1.38)	-2.53 (-11.66, 6.60)
KL grade	2.87 (-4.91, 10.65)	-5.19 (-14.11, 3.74)	0.18 (-1.07, 1.43)	-3.35 (-16.92, 10.23)	-8.29 (-23.39, 6.83)	1.48 (-0.61, 3.57)	0.36 (-1.55, 2.28)	**3.15 (0.37, 5.94)*†**	-1.05 (-3.79, 1.68)	3.77 (-6.19, 13.73)	-1.00 (-4.59, 2.58)	-2.25 (-11.57, 7.07)
WOMAC pain	**106.45 (20.57, 192.3)*†**	85.13 (-1.56, 171.8)	-8.84 (-23.80, 6.12)	-29.14 (-190.1, 131.9)	-29.68 (-190.4, 131.1)	2.19 (-19.13, 23.52)	**22.72 (2.72, 42.72)***	8.20 (-14.06, 30.46)	2.21 (-28.87, 33.29)	-28.21 (-94.58, 38.16)	-22.74 (-64.21, 18.74)	55.73 (-38.37, 149.8)
WOMAC function	**89.70 (1.10, 178.3)*†**	84.21 (-4.50, 172.9)	-4.57 (-19.76, 10.62)	15.25 (-148.8, 179.3)	-1.97 (-161.9, 158.0)	-7.92 (-28.66, 12.82)	**24.78 (5.46, 44.10)*†**	0.21 (-22.65, 22.23)	2.88 (-27.93, 33.69)	-49.87 (-111.9, 12.18)	-23.57 (-64.60, 17.45)	23.58 (-73.19, 120.3)

**p*<0.05, ***p*<0.01, † *Bonferroni-adjusted p<0.025 for cartilage loss and BMLs, p<0.05 for all other domains (Bold).*

MRI = Magnetic Resonance Imaging; % = percentage; BML = Bone Marrow Lesion; Cart = Cartilage; OST = Osteophyte; KL = Kellgren and Lawrence grading; (sf) = synovial fluid; (s) = serum; IL= interleukin; CD = cluster of differentiation; TNFα = Tumour Necrosis Factor alpha; LBP = Lipopolysaccharide Binding Protein; TLR = Toll-like receptor; vs.=versus.
